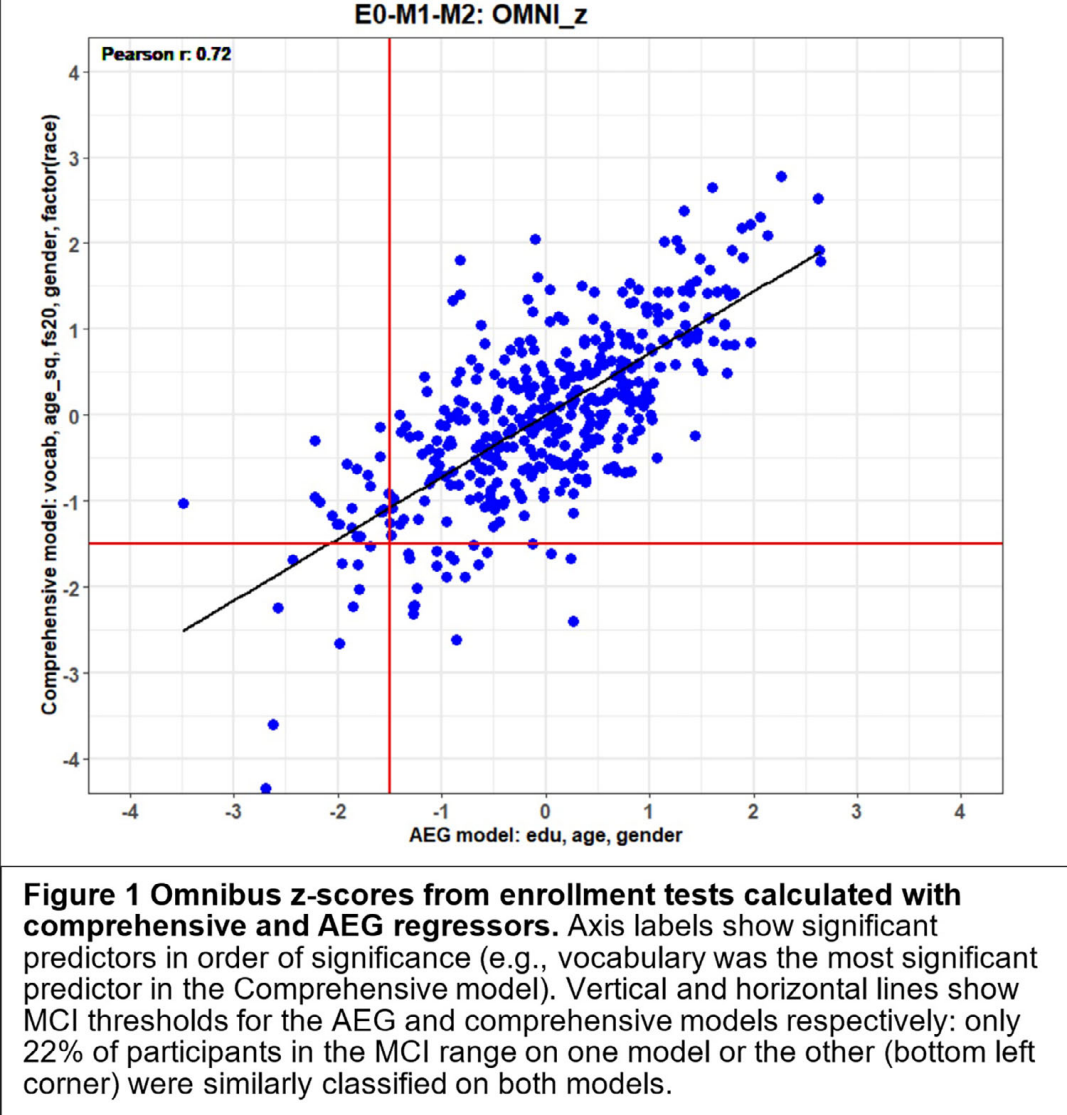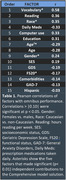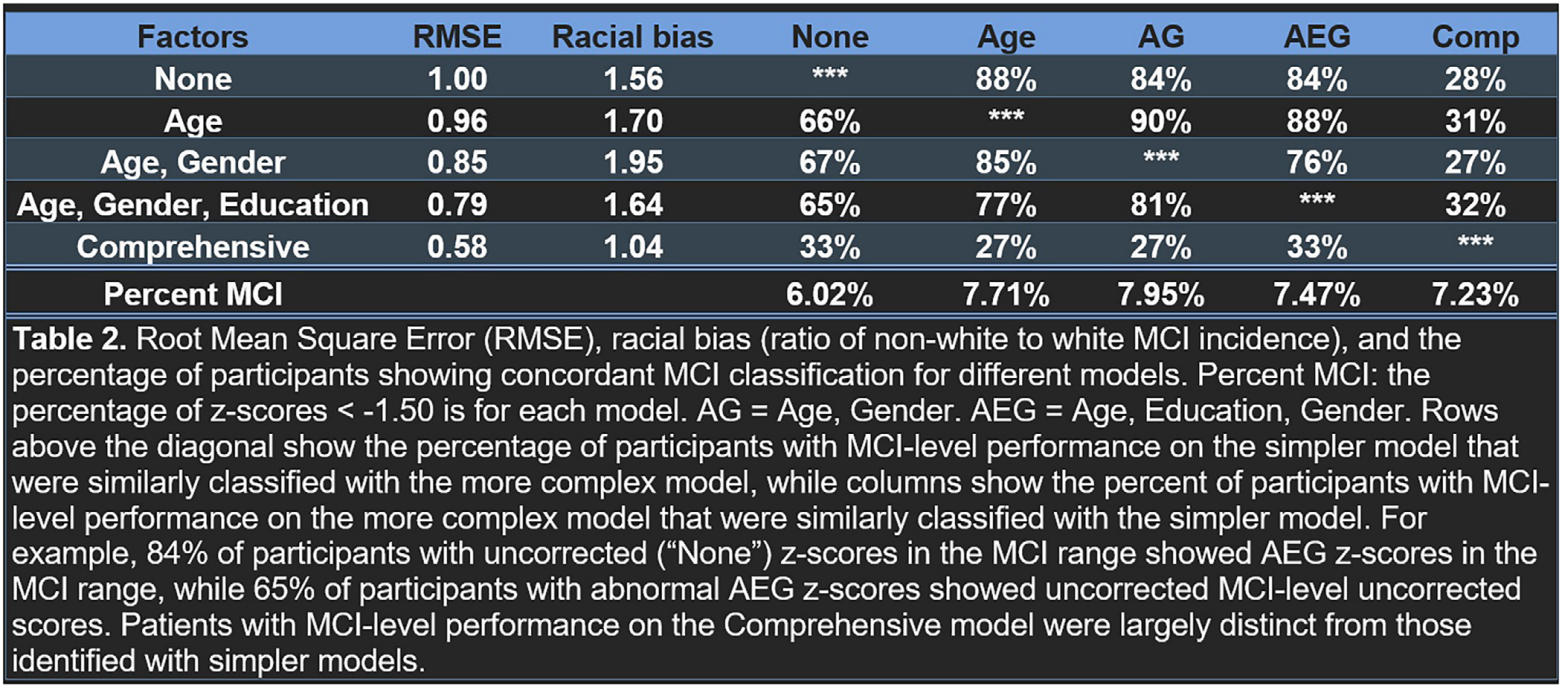# The Impact of Test‐Scoring Models on MCI Diagnosis

**DOI:** 10.1002/alz.091618

**Published:** 2025-01-03

**Authors:** David L. Woods, Juliana Baldo, Kathleen Hall, Michael Blank, Kristin Geraci, Miranda Miranda, Jas M. Chok, Sandy J. Lwi, Isabella Jaramillo, Krista Schendel, Maria G Spinelli, Timothy J Herron, David K Johnson

**Affiliations:** ^1^ Neurobehavioral Systems, Inc, Berkeley, CA USA; ^2^ Veterans Affairs Northern California Health Care System, Martinez, CA USA; ^3^ Palo Alto University, Palo Alto, CA USA; ^4^ UC Davis Alzheimer’s Disease Center, Walnut Creek, CA USA

## Abstract

**Background:**

Cognitive‐test scores in the bottom 7% of a normative distribution (z‐score < ‐1.50) are an essential component of the diagnosis of Mild Cognitive Impairment (MCI). However, different patients show MCI‐compatible performance when evaluated using scoring models that account for the influences of different demographic variables (e.g., Age only, Age and Gender, Age, Education, and Gender). Most conventional models exclude many factors (e.g., vocabulary, comorbidities, race, functional impairment, emotional status, etc.) that correlate significantly with test scores. As a result, they reduce score precision, inflate racial disparities in MCI classification, and compromise the detection of MCI in high‐functioning individuals.

**Method:**

415 older participants (mean age 70.1 years) completed three 90‐minute enrollment test sessions using the California Cognitive Assessment Battery (CCAB). Omnibus z‐scores were obtained by averaging z‐scores from 70 performance measures. Stepwise linear regression was used to identify factors that contributed significantly (p<0.01) to the solution, after outliers (typically ∼3% of scores) were eliminated. We compared MCI classification using unadjusted performance scores and models with different predictors including (1) Age, (2) Age and Gender; (3) Age, Education, and Gender (AEG), and (4) a Comprehensive model that included 15 demographic factors (see Table 1). All models were tested for heteroskedasticity, multicollinearity, and normality.

**Result:**

Table 1 shows Pearson correlations with omnibus z‐scores (averaged over test scores) of the 15 demographic factors included in the Comprehensive model. The five factors that contributed significantly (p< 0.01) and independently to the model were included in the final model solution. Figure 1 shows omnibus z‐scores calculated with AEG and Comprehensive models. Table 2 shows the model root mean squared error and the racial bias of MCI classification for each of four models, along with the percentage of patients with identical MCI classifications on the different models. Less than one‐third of participants with MCI‐level performance on the Comprehensive model showed MCI‐level performance on other models.

**Conclusion:**

MCI classification is strongly influenced by the model used to score performance. Compared to other models, a Comprehensive model accounted for more variance, reduced racial bias in MCI classification, and identified different participants with performance in the MCI range.